# miRNA-Based Early Healing Mechanism of Extraction Sockets: miR-190a-5p, a Potential Enhancer of Bone Healing

**DOI:** 10.1155/2022/7194640

**Published:** 2022-10-22

**Authors:** Shin-Kyu Lee, Su-Hyeon Jung, Sang-Jin Song, In-Gyu Lee, Jae-Yoon Choi, Homayoun Zadeh, Dong-Woon Lee, Sung-Hee Pi, Hyung-Keun You

**Affiliations:** ^1^Department of Periodontology, School of Dentistry, Wonkwang University, Iksan, Jeonbuk, Republic of Korea; ^2^Clinical Lab for Innovative Periodontology, Department of Periodontology, School of Dentistry, Wonkwang University, Iksan, Jeonbuk, Republic of Korea; ^3^VISTA Institute for Therapeutic Innovations, Woodland Hills, CA, USA

## Abstract

**Objective:**

Tooth extraction causes a wound with hard and soft tissue defects in the alveolar ridge. Few studies have reported the function of microRNAs (miRNAs) in the healing of extraction sockets. This study used bioinformatics analysis to reveal the possible relevance and role of miRNAs during the early stages following tooth extraction.

**Materials and Methods:**

Socket tissues from beagle dogs (*Canis familiaris*; two males and two females) were collected 1 and 12 hours after extraction of premolars on both sides of the mandible. miRNA expression was profiled through miRNA sequencing, and hub miRNAs showing characteristic expression patterns were selected and subjected to target enrichment analysis. Alkaline phosphatase (ALP) activity analysis and reverse transcription-quantitative polymerase chain reaction (RT-qPCR) were performed to verify the effect of hub miRNA on osteoblast differentiation and bone regeneration *in vivo*.

**Results:**

Five miRNAs were identified to have consistently high expression levels, with cfa-miR-451 showing the highest expression. Additionally, 20 hub miRNAs were selected as candidates expected to play an important role in the healing process. Pathways, such as the MAPK, axon guidance, TGF-*β*, and Wnt signaling, were significantly enriched. Among hub miRNAs, miR-190a-5p increased ALP activity and mRNA expression of osteogenic markers and increased new bone formation *in vivo*.

**Conclusions:**

Our findings suggest that miRNAs may be involved in the earliest stages of socket healing after tooth extraction and can play an important role in moderating the entire socket healing mechanism in the extraction socket.

## 1. Introduction

Tooth extraction can be performed to treat caries or periodontal disease, or for orthodontic reasons. The extraction procedure causes a wound with hard and soft tissue defects in the alveolar ridge. In a histological observation of the human extraction socket, Amler [1] reported that a blood clot was formed immediately after tooth removal and was then replaced by granulation tissue, after which the wound was gradually covered with epithelium and filled with bone. Similarly, in histological studies with dogs, blood clots were formed after tooth extraction and a gradual healing process involving granulation tissue, provisional matrix, woven bone, mineralized bone, and bone marrow was observed [2]. It has been reported that the width and height of the alveolar bone decrease after tooth extraction. For example, Johnson [3] found that most of the dimensional changes in the human extraction socket occur within the first three months of the healing process. Similarly, Schropp et al. [4] reported that two-thirds of the ridge reduction occurred within the first three months in clinical observation, continuing up to 12 months after tooth extraction. Araújo and Lindhe [5] reported that this phenomenon is a result of bundle bone resorption after tooth extraction and additional bone loss occurring for unknown reasons on the outer surface of the ridge.

As described above, the histological and clinical findings observed in the healing process of the extraction socket are well known, but the underlying biological mechanisms are not. In order to more fundamentally understand the healing process in the extraction socket, we aimed to investigate molecular mechanisms, especially those regarding microRNAs (miRNAs), which are known to regulate gene expression at the posttranscriptional stage [6]. miRNAs are composed of 19–24 nucleotides and bind directly to messenger RNAs (mRNAs) with complementary sequences, thereby controlling the expression of genes by inhibiting mRNA translation or inducing mRNA degradation in cells [7, 8]. It is known that miRNAs are involved in biological processes such as cell development, proliferation, differentiation, survival, apoptosis, and carcinogenesis by regulating numerous signaling pathways [7, 9]. In addition, miRNAs have the potential to improve tissue repair and regeneration [10] and can even control bone regeneration [11]. Chang et al. [12] reported that miR-222, a miRNA that is differentially expressed during osteoblast differentiation, can enhance *in vivo* ectopic bone formation of bone marrow-derived stem cells (BMSCs). Yang et al. [13] reported that miR-21 promotes osteoblast differentiation in BMSCs by regulating the PTEN/PI3K/Akt/HIF-1*α* pathway and improves bone regeneration in calvarial bone defects in rats.

To date, very few studies have attempted to identify the function of miRNAs in the healing process of extraction sockets. In this study, we aimed to elucidate a comprehensive molecular biological mechanism for socket healing after tooth extraction and used bioinformatics analysis to identify the function of miRNAs at the early stage after tooth extraction.

## 2. Materials and Methods

### 2.1. Experiments for Bioinformatics Analysis

#### 2.1.1. Surgical Procedure for Animal Tissue Collection

The animal research protocol was prepared before the experiment and approved by the Institutional Animal Care and Use Committee of Jeonbuk National University (approval number: JBNU 2020-0159). All procedures were conducted in compliance with the ARRIVE guidelines [14]. A total of four beagle dogs (*Canis familiaris*; two males and two females) aged about 17 months and weighing 10.48 ± 0.7319 kg were used in the experiment. Animals were purchased from a private animal experimental facility (Huvet, Iksan, Jeonbuk, Republic of Korea) and maintained under the care of a veterinarian. During the experiment, animals lived in a group kennel with indoor and outdoor spaces and were fed a soft dry pellet diet provided *ter in die* (Nutrena ultra active; Cargill Agri Purina, Seongnam, Gyeonggi, Republic of Korea). Water was available ad libitum. At the time of surgery, all four beagle dogs were deemed healthy and included in the experiment. After general anesthesia by intramuscular injection of ketamine hydrochloride (5 mg/kg; Yuhan Ketamine 50 Inj., Yuhan, Seoul, Republic of Korea) and xylazine hydrochloride (2.3 mg/kg; Rompun; Bayer Korea, Seoul, Republic of Korea), lidocaine hydrochloride (2% Lidocaine HCl & Epinephrine Injection (1 : 100,000); Yuhan) was injected into the apical mucosa of the tooth.

**Figure 1:**
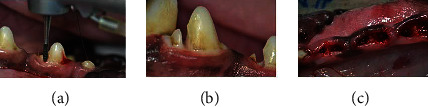
Tooth extraction process in a beagle dog. (a, b) To minimize the trauma during tooth extraction, the tooth was cut using a fissure bur in the middle of the crown to separate the root. (c) The mesial and distal portions of the tooth were carefully removed.

The second, third, and fourth premolar teeth on the left and right sides of the mandible were cut from the crown to the furcation area using a fissure bur, then extracted carefully (Figure 1). On the right side, intentional trauma was formed as a semicylindrical groove with a depth of 0.7 mm using a fissure bur at the buccal, lingual, mesial, and distal internal wall areas of the extraction socket. On the left side, care was taken to avoid any trauma apart from tooth extraction. After surgical procedures, it was confirmed that blood clots had formed normally in all extraction sockets. At 1 and 12 hours after tooth extraction, the tissue within the socket was gently scraped for sampling using a surgical curette. According to the time after tooth extraction, the wound was classified into one of four groups: 1 hour, nontrauma extraction socket (NT 1H); 1 hour, trauma extraction socket (T 1H); 12 hours, nontrauma extraction socket (NT 12H); and 12 hours, trauma extraction socket (T 12H). Researchers were blinded to group allocation, which was decided by a third party who did not directly participate in the experimental work. After the surgery procedure, animals were humanely euthanized by intravenous injection of potassium chloride (0.75 mEq/kg; Potassium Chloride Inj., JW Pharmaceutical, Seoul, Republic of Korea) after sedation with ketamine hydrochloride (5 mg/kg; Yuhan Ketamine 50 Inj., Yuhan) and xylazine hydrochloride (2.3 mg/kg; Rompun, Bayer Korea).

### 2.2. High-Throughput Sequencing of miRNAs

#### 2.2.1. RNA Isolation

Total RNA was extracted using TRIzol reagent (Invitrogen, Carlsbad, CA, USA) according to the manufacturer's protocol, and all samples of each group were pooled to collect an abundant amount of RNA. RNA quality was evaluated with an Agilent 2100 bioanalyzer (Agilent Technologies, Santa Clara, CA, USA) using an RNA 6000 Pico Chip (Agilent Technologies), and RNA quantification was performed using a NanoDrop 2000 Spectrophotometer system (Thermo Fisher Scientific, Waltham, MA, USA).

#### 2.2.2. Small RNA Library Preparation and Sequencing

According to the manufacturer's protocol, a small RNA library was constructed using the NEBNext Multiplex Small RNA Library Prep kit (New England BioLabs, Ipswich, MA, USA). A high-sensitivity DNA Assay (Agilent Technologies) was used to evaluate the yield and size distribution of the library. High-throughput sequences were produced using the single-end 75 sequencing NextSeq500 system (Illumina, San Diego, CA, USA).

## 3. Bioinformatics Analysis

### 3.1. High-Throughput Sequencing Analysis and Data Processing

Read counts mapped to 453 mature miRNA sequences of *Canis familiaris* identified in miRbase version 22 [15] were extracted. Subsequently, miRbase was used as a reference for the sequence of all miRNAs covered in this study. The expression levels were compared by log2 transformation of the data obtained by quantile normalization of read counts. miRNAs showing an expression level of less than four were considered false positives and were excluded from the analysis. A hierarchical cluster heatmap was produced using MultiExperiment Viewer [16]. Weighted gene coexpression network analysis (WGCNA) [17] was performed using the WGCNA R package to identify clusters of miRNAs with Pearson's correlation coefficients greater than 0.8 in the four groups. In the WGCNA process, at least two miRNAs were included in a single cluster, and each cluster was classified by a color name (Appendix S1, S2).

### 3.2. Screening for Hub miRNAs

A statistical analysis was performed to screen for hub miRNAs with high potential to play a role in the healing of the extraction socket. First, paired *t*-tests were performed between the following groups to identify WGCNA clusters with significant differences in mean expression level: NT 1H and NT 12H (referred to as “NT 1H-12H”), T 1H and T 12H (“T 1H-12H”), NT 1H and T 1H (“1H NT-T”), and NT 12H and T 12H (“12H NT-T”). In the miRNA clusters identified by each test, miRNAs with a fold change > 3.0 and an absolute value of Module Membership (MM) > 0.95 were selected as hub miRNAs. MM represents the correlation between each miRNA and its respective cluster [17]. The miRbase database was used to confirm whether the sequence of the selected hub miRNAs was fully conserved in humans.

### 3.3. Hub miRNA Target Prediction and Enrichment Analysis

A list of putative mRNA targets of hub miRNAs was obtained through the miRWalk 3.0 database [18]. To reduce the risk of false positives, only targets with a binding probability of 1 were selected. Pathway enrichment analysis was performed with the selected targets as follows: a two-sided hypergeometric test was applied to the term containing at least 10% or more genes in the Kyoto Encyclopedia of Genes and Genomes (KEGG) pathway database for *p* value comparison [19]. Only when the adjusted *p* value calculated by the Bonferroni step-down method was less than 0.05 was the term considered to be significantly enriched. The -log2 adjusted *p* value of each KEGG pathway was calculated, and the number of genes included in each KEGG pathway was counted. Additionally, GO term enrichment analysis was performed under the same conditions. All enrichment analyses were performed using Cytoscape version 3.8.0 for Windows with the ClueGO version 2.5.7 plugin [20].

## 4. Verification of Hub miRNA Osteogenic Effects *In Vitro*

### 4.1. Culture of Human MSCs and Transfection of miRNA Mimics or Inhibitors

Human study protocols and patient recruitment were approved by the Institutional Review Board at the Wonkwang University Dental Hospital (approval number: WKDIRB202103-01). The patients were 38–65 years of age (*n* = 3) and provided written informed consent. The three participants required tooth extractions due to caries, a cracked tooth, and orthodontic reasons and underwent the following procedure. First, local anesthesia was applied by injecting lidocaine hydrochloride (2% lidocaine HCl & epinephrine injection (1 : 100,000); Yuhan) to the apical mucosa of the tooth, and then the tooth was carefully extracted using an elevator and forceps. Dental socket-derived mesenchymal stem cells (dsMSCs) were obtained by aspirating the blood from the tooth sockets immediately after extraction using an 18-gauge needle syringe. The dsMSCs were then isolated and expanded as described previously [21]. After primary culture, subcultures were performed at intervals of 2–3 days depending on confluency. Cells were transfected with miRNA mimic or inhibitor using Lipofectamine 2000 (Invitrogen) following the manufacturer's protocol. Transfection was performed when passage number 3 cells reached 90% confluency. To validate miRNA transfection efficacy, RNA isolation was performed with TRIzol reagent (Invitrogen) and miRNA expression was evaluated using miScript Primer Assay (Qiagen, Venlo, Netherlands). According to the manufacturer's instructions, miRNAs were reverse transcribed using miScript II RT Kit (Qiagen), amplified using miScript SYBR Green PCR Kit (Qiagen), and detected using an ABI PRISM1 7300 unit (Applied Biosystems, Foster City, CA, USA). During the experiment, no supplements were provided other than fetal bovine serum in the cell culture medium. AccuTarget™ Human miRNA mimics and inhibitors (Bioneer, Daejeon, Republic of Korea; catalog numbers SMM-001, SMI-001, SMC-2001, SMC-2101) were used for the miRNA mimics, miRNA inhibitors, miRNA negative control mimic, and miRNA negative control inhibitor in the subsequent analyses.

### 4.2. Alkaline Phosphatase (ALP) Activity Assay

ALP activity was determined using the method described by Kim et al. [22], with p-nitrophenylphosphate as the substrate. ALP activity was normalized to the total protein content measured by a BCA protein assay (Thermo Fisher Scientific), according to the manufacturer's instructions. After calculating each nmol/30 min/mg of protein, the fold change for the corresponding negative control was log2 transformed to represent the data.

### 4.3. Reverse Transcription-Quantitative Polymerase Chain Reaction (RT-qPCR) Assays

Total RNA was extracted from cultured cells with TRIzol reagent (Invitrogen), according to the manufacturer's protocol, and quantified using a Nano-drop 2000 (Thermo Fisher Scientific). First-strand cDNA was synthesized using the PrimeScript™ RT Reagent Kit (Takara Bio, Otsu, Shiga, Japan). qPCR was performed using HiPi™ Real-time PCR 2× Master Mix (Elpis Biotech, Daejeon, Republic of Korea) with *GAPDH* as the internal control. To determine the expression levels of *ALP*, *collagen type I α 1 (COL1α1)*, *bone sialoprotein (BSP)*, *osteocalcin (OC)*, *osteonectin (ON)*, *osterix (OSX)*, and *runt-related transcription factor 2 (Runx2)*, the cDNA samples were analyzed by qPCR using an ABI PRISM1 7300 unit (Applied Biosystems). Primers for the genes are listed in Appendix S6.

## 5. Verification of Hub miRNA Osteogenic Effects *In Vivo*

### 5.1. Administration of a Candidate Osteogenic Hub miRNA to Dog Extraction Sockets

For histologic analysis of bone regeneration under candidate osteogenic miRNA treatment, another female beagle dog (*Canis familiaris*) aged approximately 18 months, weighing 10.25 kg, was used. Animal purchase, management, and preoperative sedation were performed following the procedures described above.

The BMSCs were isolated and expanded from humerus bone marrow as described previously [23]. Candidate osteogenic hub miRNA was transfected into dog BMSCs using the same method described above. After preparing BMSCs transfected with miRNA, the third premolar teeth on both sides of the mandible were extracted by the method described above. BMSCs transfected with miRNA detached with trypsin-EDTA solution (Trypsin-EDTA [0.05%], phenol red, Thermo Fisher Scientific) were soaked in a collagen matrix (Ateloplug, Hyundai Bioland, Cheongju, Republic of Korea) and inserted into the extraction socket of the mandibular right third premolar mesial root. On the corresponding left socket, only a collagen matrix was inserted as a control.

### 5.2. Histological Analysis

After four weeks, the animal was euthanized and samples were retrieved. After decalcification, the extraction socket was cross-sectioned, with the widest section on the buccolingual side, then subjected to Masson's trichrome staining. New bone formation was evaluated using ImageJ 1.53e for Windows (National Institutes of Health, Bethesda, MD, USA) by measuring the ratio of new bone area to a rectangular region of interest (ROI) area of a known size. New bone formation in the ROI was evaluated in each of the coronal, middle, and apical sections of the extraction socket, and the average value of the three sections was calculated to compare the experimental and control groups. Additionally, the ratio of red-colored, mineralized bone tissue and blue-colored connective tissue was also measured.

## 6. Statistical Analysis

The results of the ALP activity, RT-qPCR, and histological analysis were expressed as the mean ± standard error of the mean (SEM), and two-tailed *t*-tests were used to compare the control and experimental groups. Data were considered statistically significant at *p* < 0.05. Two-tailed *t*-tests were performed using GraphPad Prism version 7.0.0 for Windows (GraphPad Software, San Diego, CA, USA, http://www.graphpad.com).

## 7. Results

### 7.1. miRNA Expression Profiles in Extraction Socket

**Figure 2:**
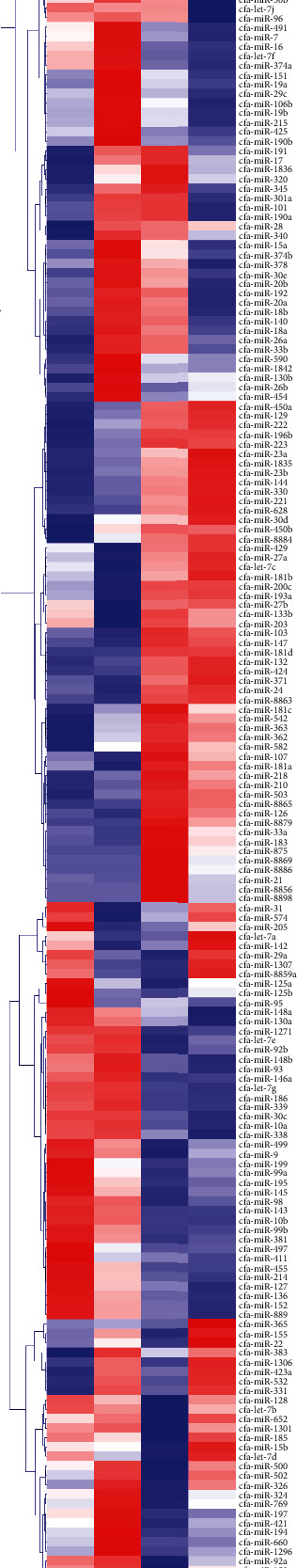
Hierarchical cluster heatmap of miRNA expression. For each miRNA, *Z*-score normalized expression level from 4 extraction socket groups was represented. A total of 188 miRNAs were divided into clusters with similar expression patterns. NT 1H: nontrauma extraction socket after 1 hour; T 1H: trauma extraction socket after 1 hour; NT 12H: nontrauma extraction socket after 12 hours; T 12H: trauma extraction socket after 12 hours.

Among 453 miRNAs of *Canis familiaris*, the expression of 188 was confirmed after excluding false positives, and clusters showing similar expression patterns were identified (Figure 2). The number of miRNAs with an expression level > 15 was 11 in NT 1H, 13 in T 1H, 9 in NT 12H, and 8 in T 12H. The number of miRNAs according to the expression level range is presented in Appendix S3. Expression was the highest for cfa-miR-451, cfa-let-7f, cfa-let-7g, cfa-miR-486, and cfa-miR-486-3p in each of the four groups (Table 1). In particular, cfa-miR-451 showed the highest expression level in all groups.

### 7.2. Selection of Hub miRNAs Expected to Regulate the Socket Healing Process

Hub miRNAs with an absolute value of MM > 0.95 and a fold change > 3 were selected according to the four comparisons described in Screening for Hub miRNAs (Table 2). At each comparison, six miRNAs were selected from NT 1H-12H, 18 from T 1H-12H, seven from 1H NT-T, and three from 12H NT-T. cfa-miR-190a and cfa-miR-301a were shown in three comparisons, and cfa-miR-18a, cfa-miR-18b, cfa-miR-33b, cfa-miR-130a, cfa-miR-221, cfa-miR-424, cfa-miR-429, cfa-miR-450a, cfa-miR-628, and cfa-miR-1835 were shown in two comparisons. cfa-miR-7, cfa-miR-19b, cfa-miR-26a, cfa-miR-29c, cfa-miR-101, cfa-miR-106b, cfa-miR-181b, and cfa-miR-215 were each identified in only one comparison. A total of 20 miRNAs were identified as potentially playing a role in the healing process of the extraction socket.

### 7.3. Signaling Pathways Identified in Target Enrichment Analysis of Hub miRNAs

**Figure 3:**
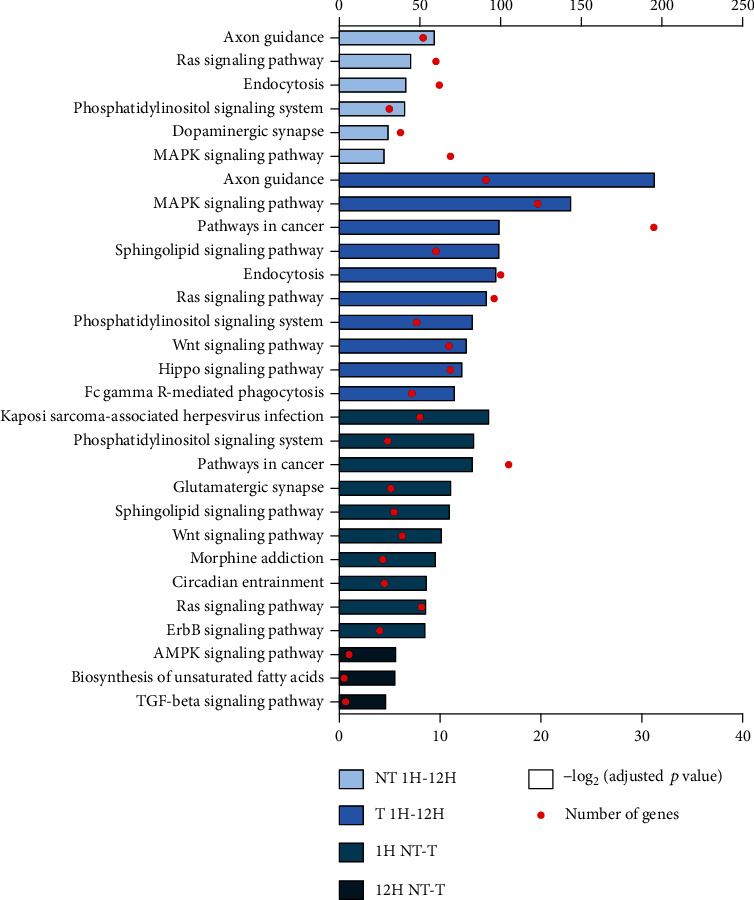
Target enrichment analysis of hub miRNAs using the KEGG pathway database. The top 10 KEGG pathways with high significance levels were plotted. The bar graph represents -log2 (adjusted *p* value) of each KEGG pathway, and the red dot represents the number of target genes belonging to each term. The number of significantly enriched signaling pathways was less than 10 in NT 1H-12H and 12H NT-T. The full list of results is shown in Appendix S4. NT 1H: nontrauma extraction socket after one hour; T 1H: trauma extraction socket after one hour; NT 12H: nontrauma extraction socket after 12 hours; T 12H: trauma extraction socket after 12 hours. NT 1H-12H: comparison between NT 1H and NT 12H; T 1H-12H: comparison between T 1H and T 12H; 1H NT-T: comparison between NT 1H and T 1H; 12H NT-T: comparison between NT 12H and T 12H.

The six miRNAs identified in NT 1H-12H targeted 3161 mRNAs, and KEGG pathway enrichment analysis revealed six significantly enriched pathways. Of these pathways, axon guidance was the most significantly enriched, and the MAPK signaling pathway contained the largest number of target genes (Figure 3, Appendix S4). Of the 18 hub miRNAs selected from T 1H-12H, 48 pathways were significantly enriched by 5329 target genes. Axon guidance was most significantly enriched, and pathways in cancer contained the largest number of target genes (Figure 3, Appendix S4). Seven hub miRNAs were selected from 1H NT-T, representing 2541 target genes and 31 significantly enriched pathways. Kaposi sarcoma-associated herpesvirus infection was the most significantly enriched pathway, and pathways in cancer contained the largest number of target genes (Figure 3, Appendix S4). The three hub miRNAs selected from 12H NT-T corresponded to 274 target genes with three significantly enriched pathways. The AMPK signaling pathway was enriched most significantly and contained the largest number of target genes (Figure 3, Appendix S4). The results of the GO term enrichment analysis are attached in Appendix S5.

### 7.4. miRNAs Selected for Osteogenic Effect Verification

Among the four groups, the most common miRNAs were cfa-miR-190a and cfa-miR-301a, which appeared in three comparisons and were always expressed when the extraction socket was traumatized (T 1H-12H, 1H NT-T, and 12H NT-T). These miRNAs were also found to be conserved in humans as miR-190a-5p and miR-301a-3p, respectively, and were therefore selected as candidates for analyzing the effect of osteoblast differentiation (Table 2).

### 7.5. miR-190a-5p Improved Osteoblast Differentiation *In Vitro* and Bone Regeneration *In Vivo*

**Figure 4:**
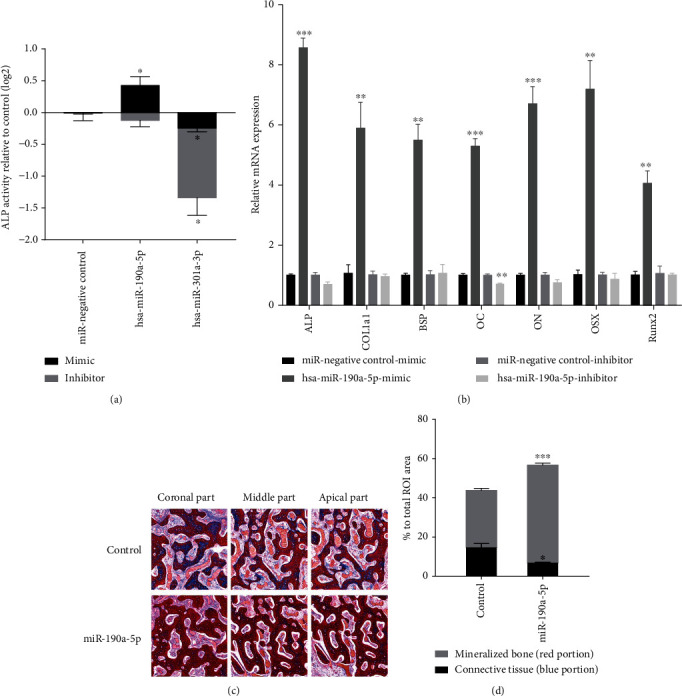
Osteogenic effects of candidate miRNAs. (a) Alkaline phosphatase (ALP) activity after transfection with miR-190a-5p, miR-301a-3p mimic, and inhibitor in human dental socket-derived mesenchymal stem cells (dsMSCs). (b) mRNA expression of osteoblast differentiation markers after transfection with the miR-190a-5p mimic and inhibitor in dsMSCs. (c) Histological findings 4 weeks after bone marrow-derived stem cells (BMSCs) transfected with miR-190a-5p were placed in the extraction socket (Masson's trichrome stain). (d) Histomorphometric analysis of the amount of mineralized new bone formation and the amount of unmineralized connective tissue after administration of miR-190a-5p. Values are expressed as the mean ± standard error of the mean (SEM). ^∗^*p* < 0.05;  ^∗∗^*p* < 0.01;  ^∗∗∗^*p* < 0.001.

In human dsMSCs, miR-190a-5p regulation and ALP activity were positively correlated (Figure 4(a)). In contrast, both miR-301a-3p upregulation and downregulation were associated with decreased ALP activity. To identify miRNA conditions that can increase bone markers, additional analysis was performed only on miR-190a-5p. In RT-qPCR analysis of osteoblast differentiation markers, miR-190a-5p mimic significantly increased the mRNA expression of *ALP*, *COL1α1*, *BSP*, *OC*, *ON*, *OSX*, and *Runx2* compared to the miRNA negative control mimic (Figure 4(b)). Conversely, miR-190a-5p inhibitor treatment decreased all osteoblast differentiation markers, except *BSP*, when compared with the miRNA negative control inhibitor. Histological analysis revealed that the degree of extraction site mineralization progressed faster under mir-190a-5p treatment than under the control. In addition, when miR-190a-5p was administered, the amount of new bone formed was significantly higher than that in the control, and the amount of nonmineralized connective tissue was significantly lower (Figures 4(c) and 4(d)).

## 8. Discussion

In the present investigation of the molecular mechanism of tooth extraction socket healing, high-throughput sequencing was performed to profile miRNAs involved in early healing, and their functions were predicted through target enrichment analysis. We identified several miRNAs with characteristic expression patterns and found that they regulated several signaling pathways that may be closely related to socket healing after tooth extraction. It was confirmed that miR-190a-5p administration enhanced osteoblast differentiation and new bone formation.

Few studies have investigated molecular biological changes in extraction sockets. In a study of in situ hybridization of mRNA in rats [24], it was reported that bone morphogenetic protein 2 (*BMP2*) was strongly expressed after two days, but rarely expressed on days 1, 3, 4, 5, 7, or 14 after extraction. Additionally, it was reported that the expression of *Runx2* and OC gradually increased at 7, 10, and 14 days in rat extraction sockets compared to three days after extraction [25]. These studies represent meaningful pioneering experiments, showing the extraction socket healing process in terms of gene expression, but their results are limited because only a small number of specific genes were studied. Recently, a study examined the expression levels of 148 myofibroblast-related mRNAs over time in rabbit extraction sockets [26], and another investigated the incomplete bone healing that occurred in the extraction sockets of mice deficient in miR-21-5p [27]. Despite these studies, few have used miRNA to observe the molecular mechanisms related to the healing process.

In the present study, cfa-miR-451, cfa-let-7f, cfa-let-7g, cfa-miR-486, and cfa-miR-486-3p were highly expressed in all experimental groups (Table 1). This suggests that these miRNAs are involved in the early phase of healing in the extraction socket. These five miRNAs correspond to miR-451a, let-7f-5p, let-7g-5p, miR-486-5p, and miR-486-3p, respectively, in humans (Table 1). Pan et al. [28] reported that blocking miR-451 in human BMSCs increased the protein stability of Runx2, thereby promoting osteoblastogenesis. High levels of expression of miR-451 may interfere with osteoblast differentiation of BMSCs in the early extraction socket. In other words, miR-451 has the potential to regulate osteogenesis in a manner that inhibits osteogenesis in the early extraction socket. let-7 was the first miRNA identified in humans and is known to be highly conserved between species [29]. This miRNA reportedly can regulate osteogenesis and bone formation [30]. let-7f and let-7g belong to the let-7 family [31]. It has been reported that let-7f-5p can regulate the survival of BMSCs [32]. In the case of miR-486-5p, it has been reported that this miRNA can mediate wound healing by promoting angiogenesis through transport from adipose-derived stem cells by extracellular vesicles [33]. Based on these previous studies, it can be inferred that these five miRNAs are highly expressed in early tooth extraction sockets and play essential roles in socket healing processes, including osteogenesis and angiogenesis. Further studies are needed to elucidate their function in the early extraction socket.

Along with the five highly expressed miRNAs in all tooth extraction socket groups, we identified 20 hub miRNAs via bioinformatics analysis likely to be involved in the extraction socket healing process. In target enrichment analysis of hub miRNAs, it was found that many signaling pathways related to the socket healing process were undergoing regulation soon after extraction (Table 2, Figure 3, Appendix S4). Among these signaling pathways, many were related to the inflammatory responses that occur during socket healing. The MAPK signaling pathway was significantly enriched in NT 1H-12H, T 1H-12H, and 1H NT-T. This pathway is involved in cell survival and proliferation, as well as various inflammatory responses, and is known to regulate migration and proliferation of keratinocytes in wounds [34, 35]. Our results suggest that the MAPK signal pathway mediates the inflammatory response and regulates the proliferation of the keratinocytes that change within the early extraction socket wound. The Ras signaling pathway, significantly enriched in NT 1H-12H, T 1H-12H, 1H NT-T, and the PI3K-Akt signaling pathway, significantly enriched in T 1H-12H, are also capable of regulating cell survival and proliferation [36, 37]. Additionally, Fc gamma R-mediated phagocytosis, significantly enriched in T 1H-12H, is known to induce IgG-related phagocytosis and mediate early inflammatory responses [38]. The hub miRNAs found in this study, therefore, appear to regulate wound healing in the socket immediately after tooth extraction via signaling pathways related to inflammatory responses, cell survival, and proliferation.

The importance of osteogenesis and angiogenesis during socket healing has been consistently emphasized; however, the importance of the nervous system in the regeneration of peripheral tissues has only recently received attention. Evidence suggests that neuropeptides secreted from sensory and autonomic nerves can play a central role in cutaneous wound healing [39, 40]. In addition, it has been reported that the central and peripheral nervous systems can regulate bone remodeling, metabolism, and hematopoietic homeostasis of the bone marrow [41], and sensory and autonomic nerves can control the migration and osteogenesis of BMSCs through several neurotransmitters [42]. In the present study, signaling pathways related to nerve function and regulation, such as axon guidance, dopaminergic synapse, cholinergic synapse, morphine addiction, GABAergic synapse, and glutamatergic synapse, were significantly enriched in T 1H-12H and 1H NT-T (Figure 3, Appendix S4). This suggests that, immediately after extraction, the healing process of the extraction socket is in some way controlled through the nervous system, likely through miRNAs pertaining to nerve regeneration and neurotransmitter control.

In addition to pathways related to wound healing, a number of pathways closely related to bone regeneration were identified in this study. The TGF-*β* signaling pathway, which was significantly enriched in T 1H-12H, 1H NT-T, and 12H NT-T, is known to regulate bone formation and homeostasis [43, 44]. The Wnt signaling pathway, which was significantly enriched in T 1H-12H and 1H NT-T, can also regulate bone homeostasis. Wnt signaling plays an important role in the osteogenic differentiation of MSCs, and Wnt factors are known therapeutic targets that can promote bone regeneration after trauma [45]. The hippo signaling pathway, which was significantly enriched in T 1H-12H, has been suggested to contribute to bone metabolism and maintenance of bone homeostasis by regulating osteogenesis and osteoclast formation [46, 47]. It is known that the PI3K-Akt and mTOR signaling pathways, which were significantly enriched in T 1H-12H, interact closely with each other to control osteoblast differentiation of MSCs by receiving signals from Wnt ligands [48]. In addition, the MAPK signaling pathway, which was significantly enriched in NT 1H-12H, T 1H-12H, and 1H NT-T, is known to affect osteoblast commitment and differentiation. Among MAPKs, ERK and p38 in particular play an essential role in bone formation [49]. These findings suggest that, although bone regeneration represents a later stage of wound healing, related signaling pathways can be regulated by miRNAs immediately after tooth extraction.

In the KEGG pathway enrichment analysis with 1H NT-T hub miRNAs, the MAPK signaling pathway, Wnt signaling pathway, TGF-*β* signaling pathway, osteoclast differentiation, glutamatergic synapse, axon guidance, GABAergic synapse, cholinergic synapse, and dopaminergic synapse were significantly enriched (Figure 3, Appendix S4). This suggests that, upon trauma to the extraction socket, altered hub miRNAs can regulate responses such as inflammation, bone homeostasis, and nerve function, at a time point of 1 h. In KEGG pathway enrichment analysis with 12H NT-T hub miRNAs, the AMPK signaling pathway and TGF-*β* signaling pathway were significantly enriched (Figure 3, Appendix S4). The AMPK signal can regulate osteoclast differentiation through RANKL [43]. This suggests that when trauma is applied to the extraction socket, the altered hub miRNAs at 12 h can regulate bone homeostasis. When the hub miRNAs were selected to reflect the fold change according to the presence of trauma (1H NT-T, 12H NT-T), the number of hub miRNAs decreased from 6 to 3 at 12 h compared to 1 h, and accordingly, the number of significantly enriched signaling pathways also decreased from 31 to 3 (Table 2, Figure 3, Appendix S4). From this, at the time point of 12 h compared to 1 h, it can be inferred that the effect of trauma applied to the extraction socket, at the miRNA level, can be reduced by the biological healing process. In addition, it is possible that various signaling pathways regulated by 1H NT-T and 12H NT-T hub miRNAs are involved in the molecular biological process for normal healing of traumatized early extraction sockets.

According to histological studies, an inflammatory stage begins approximately one day after tooth extraction. During this process, blood clots, inflammatory cells, and MSCs appear [2]. However, the results of this study imply that not only inflammatory response but also the regulation of processes such as nerve regeneration and neurotransmitter function, angiogenesis, and bone regeneration begin earlier than previously thought. It is, therefore, possible that a large time difference exists between the appearance of miRNAs and the onset of histological phenomena. This would mean that miRNAs can collectively begin to control healing immediately after trauma and, thus, are important in determining the direction of the overall healing process. If these possibilities are experimentally verified in the future, a therapeutic strategy to promote healing by regulating the function of miRNAs in the socket wound immediately after extraction will be possible. In addition, miRNAs could be regulated so that a normal healing mechanism can be achieved even when trauma occurs during extraction. Clinically, this molecular principle could be applied not only to the extraction socket but also in immediate implant placement after tooth extraction.

Furthermore, this study evaluated the potential of miR-190a-5p to improve bone regeneration in the extraction socket. miR-190a-5p may regulate bone regeneration in the early stage immediately after tooth extraction, and the underlying molecular mechanism should be elucidated in future studies. While the functions of miR-190a-5p have been studied in relation to some diseases like human cancers and diabetes mellitus [50], few previous studies have examined bone regeneration. For the potential clinical applications of miR-190a-5p, validation of the promotion of bone regeneration by miR-190a-5p in humans and verification of its adverse effects should be carried out in future studies.

## 9. Conclusions

This study profiled miRNA expression and function in the healing process at the tooth extraction socket. The study was limited in that only the sockets in the very early healing stage, at 1 and 12 hours postextraction, were analyzed. The present study implies that miRNAs may be involved in healing from the earliest stages and can play an important role in overseeing the entire wound healing mechanism in the extraction socket. Further studies that include more time points after tooth extraction will be needed to identify miRNAs with key roles in the overall wound healing process. Additionally, the relationship between the mRNA targeted by miRNAs and the related signaling pathways as they relate to the healing process must be further validated through additional research. To our knowledge, this study is the first attempt to look at the wound healing mechanism of tooth extraction sockets in terms of miRNA activity. Based on this understanding of biological healing mechanisms, it is possible to more fundamentally understand wound healing following tooth extraction. These findings are expected to have an impact on the development of clinical technologies, such as bone grafts and implant surgeries, to improve the efficiency of socket wound healing.

## Figures and Tables

**Table 1:** Top miRNAs for each extraction socket group according to expression level.

miRNA	Corresponding miRNAs in humans	NT 1H		T 1H		NT 12H		T 12H	
		Expression level	Rank	Expression level	Rank	Expression level	Rank	Expression level	Rank
cfa-miR-451	hsa-miR-451a	21.482	1	21.909	1	20.332	1	20.787	1
cfa-let-7f	hsa-let-7f-5p	17.394	2	18.018	2	16.835	2	16.643	2
cfa-let-7g	hsa-let-7g-5p	17.236	3	17.305	5	16.222	3	16.137	3
cfa-miR-486	hsa-miR-486-5p	16.822	4	17.607	4	15.546	4	16.116	4
cfa-miR-486-3p	hsa-miR-486-3p	16.814	5	17.617	3	15.527	5	16.104	5

**Table 2:** Hub miRNAs selected through bioinformatics analysis.

	Comparison of hub miRNA selection								Corresponding miRNA in humans	Fully conserved miRNA sequence in humans
miRNA	NT 1H-12H		T 1H-12H		1H NT-T		12H NT-T			
	Selection	Upregulated in	Selection	Upregulated in	Selection	Upregulated in	Selection	Upregulated in		
cfa-miR-190a	—	—	√	1H	√	T	√	NT	hsa-miR-190a-5p	O
cfa-miR-301a	—	—	√	1H	√	T	√	NT	hsa-miR-301a-3p	O
cfa-miR-18a	—	—	√	1H	√	T	—	—	hsa-miR-18a-5p	X
cfa-miR-18b	—	—	√	1H	√	T	—	—	hsa-miR-18b-5p	X
cfa-miR-33b	—	—	√	1H	√	T	—	—	hsa-miR-33b-5p	O
cfa-miR-130a	√	1H	√	1H	—	—	—	—	hsa-miR-130a-3p	O
cfa-miR-221	√	12H	√	12H	—	—	—	—	hsa-miR-221-3p	X
cfa-miR-424	√	12H	√	12H	—	—	—	—	hsa-miR-424-3p	O
cfa-miR-429	—	—	√	12H	√	NT	—	—	hsa-miR-429	X
cfa-miR-450a	√	12H	√	12H	—	—	—	—	hsa-miR-450a-5p	X
cfa-miR-628	√	12H	√	12H	—	—	—	—	hsa-miR-628-5p	O
cfa-miR-1835	√	12H	√	12H	—	—	—	—	None in humans	X
cfa-miR-7	—	—	√	1H	—	—	—	—	hsa-miR-7-5p	X
cfa-miR-19b	—	—	√	1H	—	—	—	—	hsa-miR-19b-3p	X
cfa-miR-26a	—	—	—	—	√	T	—	—	hsa-miR-26a-5p	O
cfa-miR-29c	—	—	√	1H	—	—	—	—	hsa-miR-29c-3p	O
cfa-miR-101	—	—	—	—	—	—	√	NT	hsa-miR-101-3p	X
cfa-miR-106b	—	—	√	1H	—	—	—	—	hsa-miR-106b-5p	O
cfa-miR-181b	—	—	√	12H	—	—	—	—	hsa-miR-181b-5p	X
cfa-miR-215	—	—	√	1H	—	—	—	—	hsa-miR-215-5p	X

NT 1H-12H: comparison between NT 1H and NT 12H; T 1H-12H: comparison between T 1H and T 12H; 1H NT-T: comparison between NT 1H and T 1H; 12H NT-T: comparison between NT 12H and T 12H; NT 1H: nontrauma extraction socket after 1 h; T 1H: trauma extraction socket after 1 h; NT 12H: nontrauma extraction socket after 12 h; T 12H: trauma extraction socket after 12 h. √ indicates selection as the hub miRNA in the corresponding comparison.

## Data Availability

The data that support the findings of this study are available from the corresponding author upon reasonable request.

## Supplementary Materials

Supplementary Materials Appendix S1: WGCNA process. Appendix S2: a list of modules classified as a result of weighted gene co-expression analysis. Appendix S3: the number of miRNAs according to the expression level range. Appendix S4: all signaling pathways from target enrichment analysis of hub miRNAs using the KEGG pathway database. Appendix S5: all GO terms from target enrichment analysis of hub miRNAs using the GO term database. Appendix S6: primers used for quantitative real-time PCR.
