# Supplementary material for: miRNA-Based Early Healing Mechanism of Extraction Sockets: miR-190a-5p, a Potential Enhancer of Bone Healing
Source: Biomed Res Int. 2022 Oct 22;2022:7194640. doi: 10.1155/2022/7194640 (PMC9617701; doi:10.1155/2022/7194640)
Supplement: Supplementary Materials — Appendix S1: WGCNA process. Appendix S2: a list of modules classified as a result of weighted gene co-expression analysis. Appendix S3: the number of miRNAs according to the expression level range. Appendix S4: all signaling pathways from target enrichment analysis of hub miRNAs using the KEGG pathway database. Appendix S5: all GO terms from target enrichment analysis of hub miRNAs using the GO term database. Appendix S6: primers used for quantitative real-time PCR. [file 7194640.f1.docx]

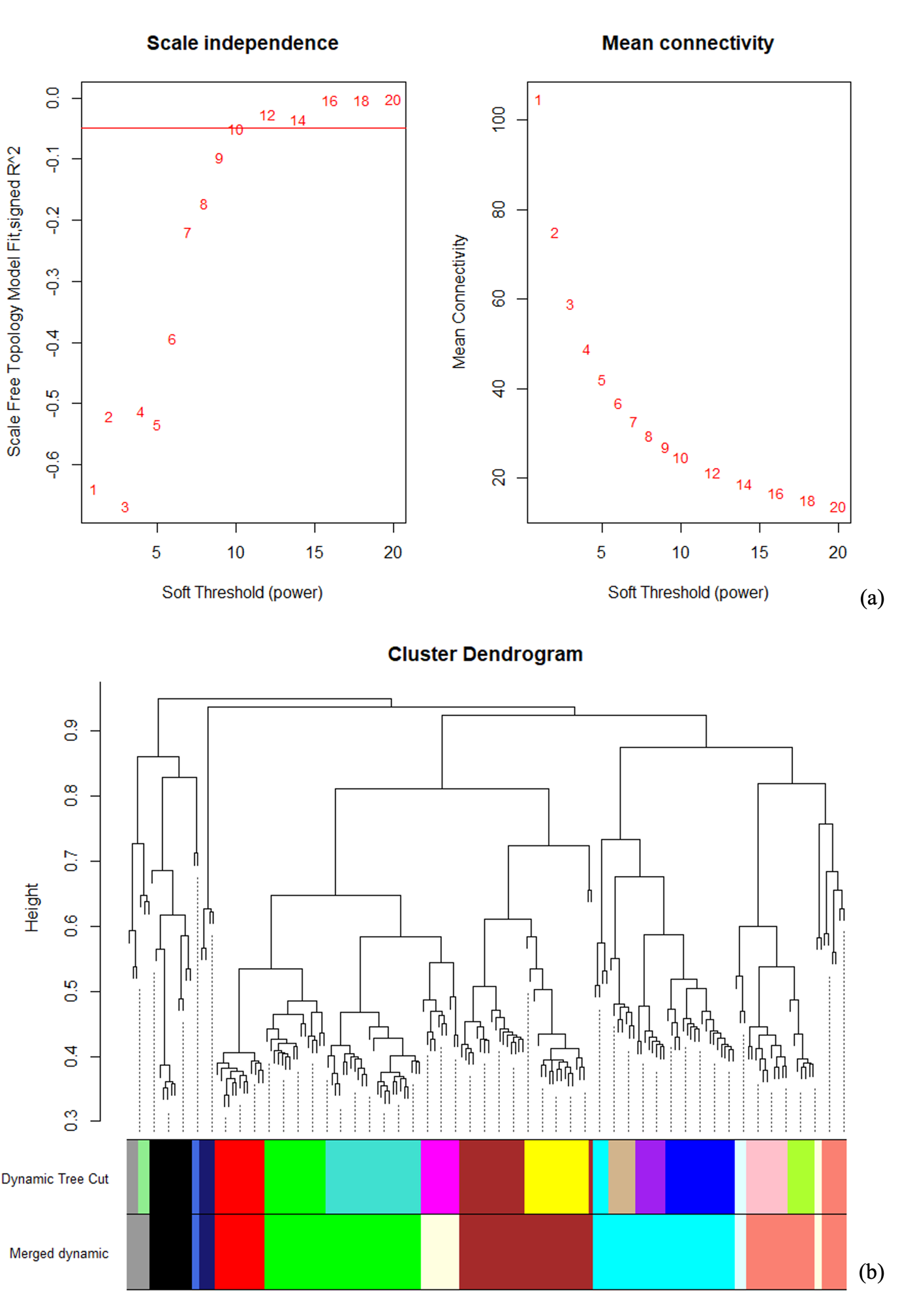


Appendix S1. WGCNA process. (a) Determination of soft-threshold power. (b) A cluster dendrogram of 188 miRNAs that passed the false positive threshold and construction of gene co-expression modules.

| Module name | miRNA count | miRNAs in the module |
| --- | --- | --- |
| Green | 41 | cfa-let-7g, cfa-miR-103, cfa-miR-10a, cfa-miR-10b, cfa-miR-127, cfa-miR-1271, cfa-miR-129, cfa-miR-136, cfa-miR-143, cfa-miR-145, cfa-miR-146a, cfa-miR-147, cfa-miR-148b, cfa-miR-152, cfa-miR-181d, cfa-miR-186, cfa-miR-195, cfa-miR-196b, cfa-miR-199, cfa-miR-214, cfa-miR-222, cfa-miR-223, cfa-miR-24, cfa-miR-30d, cfa-miR-338, cfa-miR-339, cfa-miR-362, cfa-miR-363, cfa-miR-371, cfa-miR-381, cfa-miR-411, cfa-miR-450b, cfa-miR-455, cfa-miR-497, cfa-miR-8863, cfa-miR-8865, cfa-miR-8884, cfa-miR-889, cfa-miR-93, cfa-miR-99a, cfa-miR-99b |
| Cyan | 37 | cfa-let-7c, cfa-let-7f, cfa-miR-106a, cfa-miR-106b, cfa-miR-1296, cfa-miR-130b, cfa-miR-133b, cfa-miR-151, cfa-miR-15a, cfa-miR-16, cfa-miR-181b, cfa-miR-1839, cfa-miR-1842, cfa-miR-190b, cfa-miR-193a, cfa-miR-194, cfa-miR-197, cfa-miR-19a, cfa-miR-19b, cfa-miR-200c, cfa-miR-203, cfa-miR-215, cfa-miR-26b, cfa-miR-27a, cfa-miR-27b, cfa-miR-29b, cfa-miR-29c, cfa-miR-30a, cfa-miR-374a, cfa-miR-374b, cfa-miR-425, cfa-miR-429, cfa-miR-454, cfa-miR-491, cfa-miR-590, cfa-miR-660, cfa-miR-7 |
| Brown | 35 | cfa-let-7b, cfa-let-7e, cfa-miR-107, cfa-miR-126, cfa-miR-128, cfa-miR-1301, cfa-miR-1343, cfa-miR-150, cfa-miR-181a, cfa-miR-183, cfa-miR-21, cfa-miR-25, cfa-miR-32, cfa-miR-324, cfa-miR-326, cfa-miR-33a, cfa-miR-350, cfa-miR-361, cfa-miR-421, cfa-miR-451, cfa-miR-486, cfa-miR-486-3p, cfa-miR-500, cfa-miR-502, cfa-miR-652, cfa-miR-6529, cfa-miR-769, cfa-miR-875, cfa-miR-8856, cfa-miR-8869, cfa-miR-8879, cfa-miR-8886, cfa-miR-8898, cfa-miR-92a, cfa-miR-92b |
| Salmon | 24 | cfa-miR-101, cfa-miR-125b, cfa-miR-1307, cfa-miR-140, cfa-miR-17, cfa-miR-18a, cfa-miR-18b, cfa-miR-190a, cfa-miR-191, cfa-miR-192, cfa-miR-205, cfa-miR-20a, cfa-miR-20b, cfa-miR-26a, cfa-miR-28, cfa-miR-29a, cfa-miR-301a, cfa-miR-30e, cfa-miR-31, cfa-miR-33b, cfa-miR-340, cfa-miR-345, cfa-miR-574, cfa-miR-8859a |
| Red | 13 | cfa-miR-130a, cfa-miR-132, cfa-miR-144, cfa-miR-148a, cfa-miR-1835, cfa-miR-221, cfa-miR-23a, cfa-miR-23b, cfa-miR-30c, cfa-miR-330, cfa-miR-424, cfa-miR-450a, cfa-miR-628 |
| Light yellow | 12 | cfa-miR-125a, cfa-miR-181c, cfa-miR-1836, cfa-miR-210, cfa-miR-218, cfa-miR-320, cfa-miR-499, cfa-miR-503, cfa-miR-542, cfa-miR-582, cfa-miR-9, cfa-miR-98 |
| Black | 11 | cfa-miR-1, cfa-miR-1306, cfa-miR-155, cfa-miR-184, cfa-miR-206, cfa-miR-22, cfa-miR-331, cfa-miR-379, cfa-miR-423a, cfa-miR-532, cfa-miR-708 |
| Grey60 | 6 | cfa-let-7j, cfa-miR-146b, cfa-miR-30b, cfa-miR-342, cfa-miR-365, cfa-miR-96 |
| Midnight blue | 4 | cfa-let-7d, cfa-miR-15b, cfa-miR-182, cfa-miR-185 |
| Light cyan | 3 | cfa-let-7a, cfa-miR-142, cfa-miR-378 |
| Royal blue | 2 | cfa-miR-383, cfa-miR-95 |
| Total | 188 |  |

Appendix S2. A list of modules classified as a result of weighted gene co-expression analysis. Clusters with a Pearson correlation coefficient greater than 0.8 were classified into color-named modules. At least two miRNAs were included in each module.


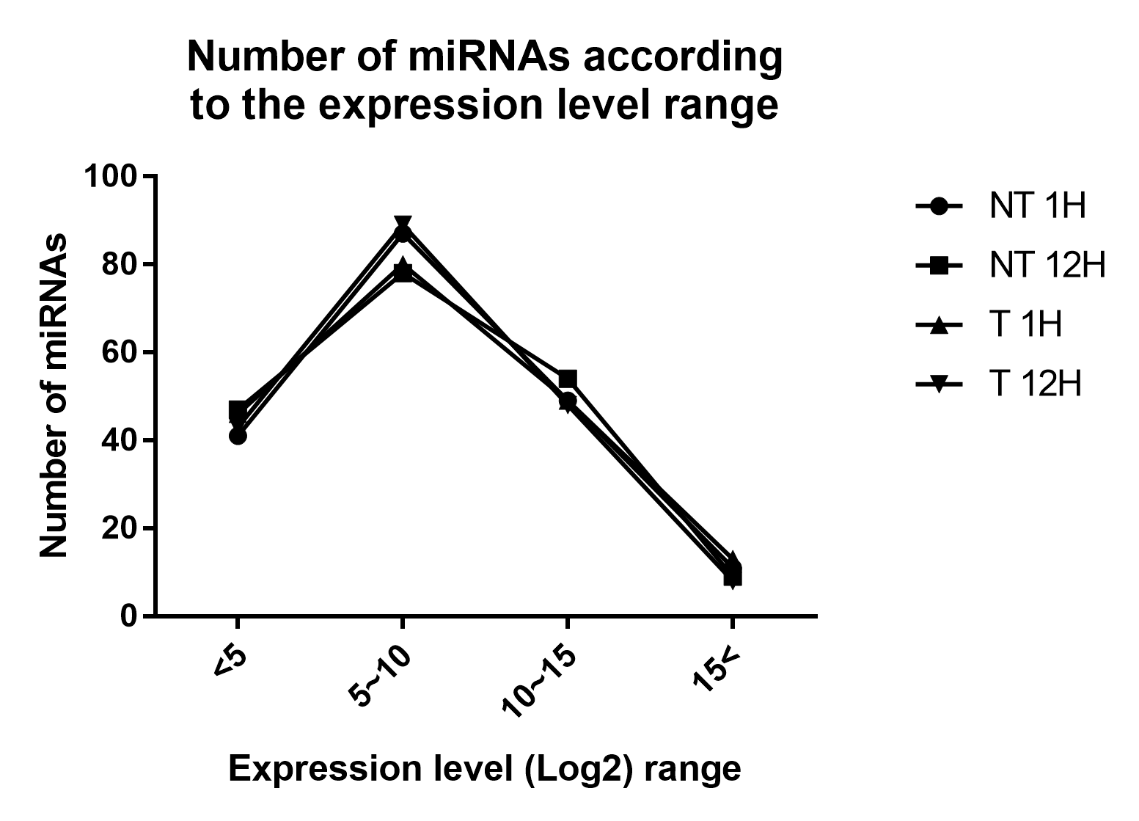


Appendix S3. The number of miRNAs according to the expression level range. NT 1H: non-trauma extraction socket after 1 hour; T 1H: trauma extraction socket after 1 hour; NT 12H: non-trauma extraction socket after 12 hours; T 12H: trauma extraction socket after 12 hours.

| KEGG pathway | –log2(adjusted *p*-value) | Number of Genes | Comparison criteria |
| --- | --- | --- | --- |
| Axon guidance | 9.448545 | 52 | NT 1H-12H |
| Ras signaling pathway | 7.063585 | 60 | NT 1H-12H |
| Endocytosis | 6.617221 | 62 | NT 1H-12H |
| Phosphatidylinositol signaling system | 6.492632 | 31 | NT 1H-12H |
| Dopaminergic synapse | 4.828416 | 38 | NT 1H-12H |
| MAPK signaling pathway | 4.441729 | 69 | NT 1H-12H |
| Axon guidance | 31.24065 | 91 | T 1H-12H |
| MAPK signaling pathway | 22.94571 | 123 | T 1H-12H |
| Pathways in cancer | 15.85598 | 195 | T 1H-12H |
| Sphingolipid signaling pathway | 15.83003 | 60 | T 1H-12H |
| Endocytosis | 15.52242 | 100 | T 1H-12H |
| Ras signaling pathway | 14.58777 | 96 | T 1H-12H |
| Phosphatidylinositol signaling system | 13.21861 | 48 | T 1H-12H |
| Wnt signaling pathway | 12.62202 | 68 | T 1H-12H |
| Hippo signaling pathway | 12.14935 | 69 | T 1H-12H |
| Fc gamma R-mediated phagocytosis | 11.43244 | 45 | T 1H-12H |
| Yersinia infection | 10.80367 | 58 | T 1H-12H |
| Dopaminergic synapse | 10.28691 | 59 | T 1H-12H |
| Cholinergic synapse | 10.09073 | 52 | T 1H-12H |
| Morphine addiction | 9.409021 | 44 | T 1H-12H |
| FoxO signaling pathway | 8.930509 | 56 | T 1H-12H |
| GABAergic synapse | 8.648667 | 43 | T 1H-12H |
| Regulation of actin cytoskeleton | 8.626569 | 86 | T 1H-12H |
| Human immunodeficiency virus 1 infection | 8.514879 | 84 | T 1H-12H |
| Choline metabolism in cancer | 8.111305 | 46 | T 1H-12H |
| Oxytocin signaling pathway | 7.960435 | 64 | T 1H-12H |
| Glutamatergic synapse | 7.959673 | 50 | T 1H-12H |
| Circadian rhythm | 7.956117 | 19 | T 1H-12H |
| Hedgehog signaling pathway | 7.955986 | 26 | T 1H-12H |
| Proteoglycans in cancer | 7.653233 | 82 | T 1H-12H |
| Autophagy | 7.631946 | 59 | T 1H-12H |
| Calcium signaling pathway | 7.546795 | 80 | T 1H-12H |
| Growth hormone synthesis, secretion and action | 7.473915 | 51 | T 1H-12H |
| ErbB signaling pathway | 7.276405 | 40 | T 1H-12H |
| Signaling pathways regulating pluripotency of stem cells | 7.256394 | 60 | T 1H-12H |
| Rap1 signaling pathway | 6.891019 | 83 | T 1H-12H |
| Thyroid hormone signaling pathway | 6.563691 | 51 | T 1H-12H |
| Inflammatory mediator regulation of TRP channels | 6.261631 | 45 | T 1H-12H |
| cAMP signaling pathway | 6.071263 | 81 | T 1H-12H |
| Long-term depression | 5.821952 | 30 | T 1H-12H |
| Nicotine addiction | 5.648717 | 22 | T 1H-12H |
| Relaxin signaling pathway | 5.322445 | 56 | T 1H-12H |
| TGF-beta signaling pathway | 5.200197 | 41 | T 1H-12H |
| Circadian entrainment | 5.162789 | 43 | T 1H-12H |
| Hepatitis B | 5.067448 | 67 | T 1H-12H |
| Insulin resistance | 4.947774 | 46 | T 1H-12H |
| Adherens junction | 4.616313 | 33 | T 1H-12H |
| Insulin signaling pathway | 4.610619 | 56 | T 1H-12H |
| Cellular senescence | 4.534738 | 61 | T 1H-12H |
| PI3K-Akt signaling pathway | 4.467207 | 127 | T 1H-12H |
| AMPK signaling pathway | 4.391269 | 50 | T 1H-12H |
| mTOR signaling pathway | 4.386366 | 62 | T 1H-12H |
| Glycerophospholipid metabolism | 4.353633 | 41 | T 1H-12H |
| Cell adhesion molecules | 4.352734 | 57 | T 1H-12H |
| Kaposi sarcoma-associated herpesvirus infection | 14.82516 | 50 | 1H NT-T |
| Phosphatidylinositol signaling system | 13.32577 | 30 | 1H NT-T |
| Pathways in cancer | 13.20647 | 105 | 1H NT-T |
| Glutamatergic synapse | 11.05001 | 32 | 1H NT-T |
| Sphingolipid signaling pathway | 10.90951 | 34 | 1H NT-T |
| Wnt signaling pathway | 10.12209 | 39 | 1H NT-T |
| Morphine addiction | 9.541472 | 27 | 1H NT-T |
| Circadian entrainment | 8.643182 | 28 | 1H NT-T |
| Ras signaling pathway | 8.562962 | 51 | 1H NT-T |
| ErbB signaling pathway | 8.500894 | 25 | 1H NT-T |
| Axon guidance | 7.743312 | 42 | 1H NT-T |
| GABAergic synapse | 7.723593 | 26 | 1H NT-T |
| Oxytocin signaling pathway | 7.487522 | 37 | 1H NT-T |
| Choline metabolism in cancer | 7.094738 | 27 | 1H NT-T |
| GnRH secretion | 6.731841 | 20 | 1H NT-T |
| Cholinergic synapse | 6.476708 | 29 | 1H NT-T |
| Long-term potentiation | 6.064399 | 20 | 1H NT-T |
| Signaling pathways regulating pluripotency of stem cells | 6.062408 | 34 | 1H NT-T |
| Phospholipase D signaling pathway | 6.05696 | 36 | 1H NT-T |
| TGF-beta signaling pathway | 6.056281 | 25 | 1H NT-T |
| GnRH signaling pathway | 5.411736 | 24 | 1H NT-T |
| Growth hormone synthesis, secretion and action | 5.397735 | 29 | 1H NT-T |
| Melanogenesis | 5.196073 | 26 | 1H NT-T |
| MAPK signaling pathway | 5.165775 | 57 | 1H NT-T |
| cAMP signaling pathway | 5.163458 | 45 | 1H NT-T |
| Long-term depression | 5.058714 | 18 | 1H NT-T |
| Yersinia infection | 5.032931 | 31 | 1H NT-T |
| Influenza A | 4.826714 | 38 | 1H NT-T |
| Dopaminergic synapse | 4.554667 | 31 | 1H NT-T |
| Osteoclast differentiation | 4.491613 | 30 | 1H NT-T |
| Measles | 4.458345 | 33 | 1H NT-T |
| AMPK signaling pathway | 5.602037 | 6 | 12H NT-T |
| Biosynthesis of unsaturated fatty acids | 5.510548 | 3 | 12H NT-T |
| TGF-beta signaling pathway | 4.60694 | 4 | 12H NT-T |

Appendix S4. All signaling pathways from target enrichment analysis of hub miRNAs using the KEGG pathway database. The -log2 (adjusted *p*-value) of each KEGG pathway and the number of genes included in the term are indicated. NT 1H: non-trauma extraction socket after 1 hour; T 1H: trauma extraction socket after 1 hour; NT 12H: non-trauma extraction socket after 12 hours; T 12H: trauma extraction socket after 12 hours. NT 1H-12H: comparison between NT 1H and NT 12H; T 1H-12H: comparison between T 1H and T 12H; 1H NT-T: comparison between NT 1H and T 1H; 12H NT-T: comparison between NT 12H and T 12H.

| Category | GO Term | –log2(adjusted *p*-value) | Number of Genes | Comparison criteria |
| --- | --- | --- | --- | --- |
| Biological process | negative regulation of biological process | 11.52943297 | 582.00 | NT 1H-12H |
|  | protein modification process | 9.918797781 | 476.00 | NT 1H-12H |
|  | macromolecule modification | 8.686970986 | 493.00 | NT 1H-12H |
|  | positive regulation of biological process | 7.95919007 | 667.00 | NT 1H-12H |
|  | negative regulation of cellular process | 7.16136551 | 526.00 | NT 1H-12H |
|  | cellular macromolecule metabolic process | 6.2286799 | 875.00 | NT 1H-12H |
|  | positive regulation of metabolic process | 5.873588234 | 451.00 | NT 1H-12H |
|  | positive regulation of macromolecule metabolic process | 4.965717709 | 422.00 | NT 1H-12H |
|  | negative regulation of intracellular signal transduction | 4.810105783 | 77.00 | NT 1H-12H |
|  | macromolecule metabolic process | 4.497008116 | 1006.00 | NT 1H-12H |
|  | regulation of metabolic process | 4.405739873 | 727.00 | NT 1H-12H |
| Cellular component | membrane-bounded organelle | 23.37294454 | 1192.00 | NT 1H-12H |
|  | intracellular membrane-bounded organelle | 19.75333738 | 1110.00 | NT 1H-12H |
|  | intracellular organelle | 18.18245936 | 1290.00 | NT 1H-12H |
|  | nucleus | 7.674930384 | 751.00 | NT 1H-12H |
|  | nucleoplasm | 5.807210249 | 400.00 | NT 1H-12H |
|  | Golgi apparatus | 5.786743565 | 171.00 | NT 1H-12H |
| Molecular function | DNA-binding transcription activator activity, RNA polymerase II-specific | 5.649212934 | 81.00 | NT 1H-12H |
|  | DNA-binding transcription activator activity | 5.572128111 | 81.00 | NT 1H-12H |
|  | DNA-binding transcription factor activity, RNA polymerase II-specific | 4.820304384 | 154.00 | NT 1H-12H |
|  | phosphotransferase activity, alcohol group as acceptor | 4.518793554 | 186.00 | NT 1H-12H |
| Biological process | positive regulation of biological process | 16.84882951 | 1057.00 | T 1H-12H |
|  | negative regulation of biological process | 14.60664987 | 901.00 | T 1H-12H |
|  | positive regulation of metabolic process | 12.60330704 | 711.00 | T 1H-12H |
|  | negative regulation of cellular process | 10.63621373 | 819.00 | T 1H-12H |
|  | regulation of metabolic process | 10.56339507 | 1150.00 | T 1H-12H |
|  | protein modification process | 10.12703436 | 728.00 | T 1H-12H |
|  | macromolecule modification | 10.06506064 | 759.00 | T 1H-12H |
|  | positive regulation of cellular process | 9.927581296 | 961.00 | T 1H-12H |
|  | positive regulation of macromolecule metabolic process | 9.68810468 | 661.00 | T 1H-12H |
|  | nervous system process | 8.654225795 | 164.00 | T 1H-12H |
|  | cellular macromolecule metabolic process | 8.511135677 | 1370.00 | T 1H-12H |
|  | macromolecule metabolic process | 7.362733251 | 1583.00 | T 1H-12H |
|  | regulation of macromolecule metabolic process | 7.201141421 | 1067.00 | T 1H-12H |
|  | positive regulation of cellular metabolic process | 6.800911363 | 624.00 | T 1H-12H |
|  | positive regulation of gene expression | 6.16091686 | 449.00 | T 1H-12H |
|  | positive regulation of nitrogen compound metabolic process | 5.244729855 | 592.00 | T 1H-12H |
|  | regulation of cellular process | 5.056585895 | 1791.00 | T 1H-12H |
|  | G protein-coupled receptor signaling pathway | 4.662586615 | 133.00 | T 1H-12H |
| Cellular component | membrane-bounded organelle | 40.49071198 | 1882.00 | T 1H-12H |
|  | intracellular membrane-bounded organelle | 32.25196689 | 1747.00 | T 1H-12H |
|  | intracellular organelle | 24.77147618 | 2021.00 | T 1H-12H |
|  | nucleus | 16.13713879 | 1188.00 | T 1H-12H |
|  | transferase complex | 14.49458517 | 180.00 | T 1H-12H |
|  | endosome | 12.8298642 | 170.00 | T 1H-12H |
|  | Golgi apparatus | 12.69608885 | 270.00 | T 1H-12H |
|  | extracellular space | 12.35015552 | 158.00 | T 1H-12H |
|  | bounding membrane of organelle | 11.34414133 | 258.00 | T 1H-12H |
|  | nucleoplasm | 11.13577162 | 628.00 | T 1H-12H |
|  | whole membrane | 8.645292723 | 222.00 | T 1H-12H |
|  | nuclear lumen | 7.992916769 | 749.00 | T 1H-12H |
|  | organelle subcompartment | 5.831764781 | 73.00 | T 1H-12H |
|  | vesicle | 4.924000285 | 336.00 | T 1H-12H |
|  | Golgi apparatus subcompartment | 4.922588034 | 67.00 | T 1H-12H |
|  | organelle lumen | 4.647442483 | 790.00 | T 1H-12H |
| Molecular function | RNA polymerase II cis-regulatory region sequence-specific DNA binding | 5.183948187 | 197.00 | T 1H-12H |
|  | kinase activity | 4.46408939 | 300.00 | T 1H-12H |
|  | cis-regulatory region sequence-specific DNA binding | 4.335300779 | 200.00 | T 1H-12H |
| Biological process | vesicle-mediated transport | 7.40395371 | 160.00 | 1H NT-T |
|  | regulation of relaxation of muscle | 4.378937016 | 5.00 | 1H NT-T |
| Cellular component | Golgi apparatus subcompartment | 10.09753058 | 42.00 | 1H NT-T |
|  | organelle subcompartment | 8.834212178 | 44.00 | 1H NT-T |
|  | transferase complex | 7.421599551 | 92.00 | 1H NT-T |
|  | trans-Golgi network | 6.90641915 | 32.00 | 1H NT-T |
|  | endosome | 5.160435961 | 85.00 | 1H NT-T |
| Molecular function | C4-dicarboxylate transmembrane transporter activity | 5.077276875 | 6.00 | 1H NT-T |
| Biological process | response to epinephrine | 12.82424337 | 3.00 | 12H NT-T |
|  | cellular response to epinephrine stimulus | 12.82424337 | 3.00 | 12H NT-T |
|  | negative regulation of protein localization to nucleus | 8.923522476 | 3.00 | 12H NT-T |

Appendix S5. All GO terms from target enrichment analysis of hub miRNAs using the GO term database. The -log2 (adjusted *p*-value) of each GO term and the number of genes included in the term are indicated. NT 1H: non-trauma extraction socket after 1 hour; T 1H: trauma extraction socket after 1 hour; NT 12H: non-trauma extraction socket after 12 hours; T 12H: trauma extraction socket after 12 hours. NT 1H-12H: comparison between NT 1H and NT 12H; T 1H-12H: comparison between T 1H and T 12H; 1H NT-T: comparison between NT 1H and T 1H; 12H NT-T: comparison between NT 12H and T 12H.

| Gene | Sequence | |
| --- | --- | --- |
|  | Forward primer | Reverse primer |
| *ALP* | TGCAGTACGAGCTGAACAGG | CGTGGTCAATTCTGCCTCCT |
| *COL1α1* | TAAAGGGTCACCGTGGCTTC | AGTCCATCTTTGCCAGGAGC |
| *BSP* | TCAGCATTTTGGGAATGGCC | GAGGTTGTTGTCTTCGAGGT |
| *OC* | CGCTACCTGTATCAATGGCTGG | CTCCTGAAAGCCGATGTGGTCA |
| *ON* | CAAGAAGCCCTGCCTGATGA | TCTTCGGTTTCCTCTGCACC |
| *OSX* | TAATGGGCTCCTTTCACCTG | CACTGGGCAGACAGTCAGAA |
| *Runx2* | CGAATGGCAGCACGCTATTAA | GTCGCCAAACAGATTCATCCA |
| *GAPDH* | GTCAGTGGTGGACCTGACCT | AGGGGAGATTCAGTGTGGTG |

Appendix S6. Primers used for quantitative real-time PCR.
